# Label-Free Liquid Chromatography–Mass Spectrometry Proteomic Analysis of the Urinary Proteome for Measuring the Escitalopram Treatment Response From Major Depressive Disorder

**DOI:** 10.3389/fpsyt.2021.700149

**Published:** 2021-09-30

**Authors:** Yuhang Huan, Jing Wei, Jingjing Zhou, Min Liu, Jian Yang, Youhe Gao

**Affiliations:** ^1^Department of Biochemistry and Molecular Biology, Gene Engineering Drug and Biotechnology Beijing Key Laboratory, Beijing Normal University, Beijing, China; ^2^The National Clinical Research Center for Mental Disorders and Beijing Key Laboratory of Mental Disorders, Beijing Anding Hospital, Capital Medical University, Beijing, China; ^3^Advanced Innovation Center for Human Brain Protection, Capital Medical University, Beijing, China

**Keywords:** major depressive disorder (MDD), treatment response biomarkers, proteomics, urine, escitalopram

## Abstract

Major depressive disorder (MDD) is a common mental disorder that can cause substantial impairments in quality of life. Clinical treatment is usually built on a trial-and-error method, which lasts ~12 weeks to evaluate whether the treatment is efficient, thereby leading to some inefficient treatment measures. Therefore, we intended to identify early candidate urine biomarkers to predict efficient treatment response in MDD patients. In this study, urine samples were collected twice from 19 respondent and 10 non-respondent MDD patients receiving 0-, 2-, and 12-week treatments with escitalopram. Differential urinary proteins were subsequently analyzed by liquid chromatography coupled with tandem mass spectrometry (LC-MS/MS). Our two pilot tests suggested that the urine proteome reflects changes associated with major depressive disorder at the early stage of treatment measures. On week 2, 20 differential proteins were identified in the response group compared with week 0, with 14 of these proteins being associated with the mechanisms of MDD. In the non-response group, 60 differential proteins were identified at week 2, with 28 of these proteins being associated with the mechanisms of MDD. In addition, differential urinary proteins at week 2 between the response and non-response groups can be clearly distinguished by using orthogonal projection on latent structure-discriminant analysis (OPLS-DA). Our small pilot tests indicated that the urine proteome can reflect early effects of escitalopram therapy between the response and non-response groups since at week 2, which may provide potential early candidate urine biomarkers to predict efficient treatment measures in MDD patients.

## Introduction

Major depressive disorder (MDD) is a severe and debilitating psychiatric disorder characterized by depressed mood, anhedonia, and altered cognitive function (1). MDD was recently ranked as the single largest contributor to global disability according to the World Health Organization (WHO) for its significant social and financial burden (2). Unfortunately, the pathogenesis has not been clearly elucidated because depression is highly heterogeneous and involves complex interactions between genetic factors and multiple molecular pathways. Moreover, the illness remains both underdiagnosed and undertreated (3). Currently, accurate diagnosis is achieved via detailed histories, mental status and physical examinations, and laboratory tests. Additionally, only partially effective trial-and-error methods are applied for next-step treatment selection. Even positive treatment effects may be very slow to appear and each level of treatment requires 12 weeks. The problem of treatment-resistant depression has led to the continued search for effective measurements for antidepressant treatments. Therefore, treatment response biomarkers are urgently needed to eliminate multiple treatment steps and provide effective treatment options.

Biomarkers are measurable changes in an individual that represent indicators of a disease state or pharmacologic response to therapeutic intervention, and they are determined by hormone measurements, genome variations, protein measurements or even neuroimaging changes. Mass spectrometry (MS) is a comprehensive unbiased method of proteomic analysis. The application of proteomics to cerebrospinal fluid (CSF), plasma and urine has significantly accelerated unbiased and high-throughput searches for potential biomarkers of nervous system diseases. Urine is not affected by homeostasis mechanisms, and subtle changes in urine are accumulated in the blood (4). Urine could also be the most convenient and amenable substance for use in MDD diagnostic or predictive tests and thus can be used as an ideal source of biomarkers to replace blood or CSF. However, urinary protein biomarkers are less explored in MDD. Further studies utilizing MS and proteomic analysis in MDD may help solidify and establish urine biomarkers for use in diagnosing and understanding the disorder and identifying therapeutic intervention responses.

Selective serotonin reuptake inhibitors (SSRIs) are widely used to treat depression due to their low toxicity and low side-effect profiles. Escitalopram is the pure S-enantiomer of racemic citalopram and inhibits the serotonin transporter protein (SERT), and it augments the serotonergic activity in the central nervous system (CNS) by binding to SERT selectively to inhibit 5-HT reuptake (5–7). However, the cumulative rate of remission was 67% after several levels of therapy, which indicates that the current treatment options are not very effective (8, 9). Further objective biomarkers that facilitate a convenient and inexpensive predictive test for depression treatment response are urgently required for clinical practice.

In this study, we performed urinary proteomic profiling to identify urine candidate biomarkers in respondent and non-respondent escitalopram MDD patients. The proteomes of the respondents and non-respondents at baseline of MDD diagnosis and 2 weeks and 12 weeks of treatment by escitalopram were analyzed using label-free high-performance liquid chromatography-tandem mass spectrometry (LC-MS/MS). The first trial aimed to identify whether urine can reflect changes associated with escitalopram treatment at the very early stage. The second trial aimed to test whether urine can distinguish different degrees of response to escitalopram from MDD patients. Our two pilot studies suggested that the urine proteome reflects changes associated with major depressive disorder at the early stage of treatment measures. Differential urinary proteins at week 2 between the response and non-response groups can be clearly distinguished, which may provide valuable clues to guide future treatment decision making.

## Materials and Methods

### Study Design and Participants

This study was approved by the Human Research and Ethics Committee of Beijing Anding Hospital (#2017-24), and written informed consent was provided by all participants. In brief, the participants were recruited from Beijing Anding Hospital and samples were taken from June to December 2018. Patients with primary psychotic disorders, organic brain disease or other chronic diseases were excluded. In the first trial, 27 samples from nine respondent subjects were analyzed to identify whether urine can reflect the treatment response at the early stage of treatment. In the second trial, 60 samples from 10 respondent patients and 10 non-respondent patients to escitalopram were analyzed to identify whether urine can distinguish different treatment responses at the very early stage of treatment. In total, 87 samples were analyzed via LC-MS/MS: 27 urine samples from nine patients in the first trial and 60 urine samples from 20 patients in the second trial.

### Diagnostic Evaluations

Demographic data for the participants, including age, sex, height and weight, time of first depressive episode and age of first onset, were collected. Exclusion criteria included mental disorders (including anxiety disorders, schizophrenia, nicotine dependence and use, alcohol dependence, and other substance abuse), serious medical conditions (such as cancer and diabetes), organic brain disease, and pregnancy or breastfeeding. The Hamilton Depression-17 Scale (HAMD-17), 16-item Quick Inventory of Depressive Symptomatology-Self-Report (QIDS-SR), YMRS and Patient Health Questionnaire-9 (PHQ-9) were administered to all patients to achieve the diagnosis at baseline, week 4, week 8, and week 12. Drug-free patients who met the DSMIV-TR criteria for major depressive episodes with a HAMD score >17 points were recruited in our study. Respondent patients were defined as the HAMD-17 scores decreased more than half than baseline at the end of the 12-weeks treatment. In addition, the QIDS-SR (assess the severity of depressive symptoms in patients with MDD or bipolar disorder completed by patients themselves) (10), the YMRS (an 11-item clinician-administered instrument used to assess the severity of mania) and the PHQ-9 (a nine-item questionnaire designed to screen for depression in primary care and other medical settings) (11) were also applied for diagnosis reference.

### Urinary Sample Processing and LC-MS/MS Analysis

Urine samples were collected at the diagnostic baseline and at 2 weeks and 12 weeks of escitalopram treatment. After collection, all of the urine samples including 27 from the first pilot study and 60 from the second pilot study were processed following the same method as follows. Urine samples were centrifuged at 3,000 × g for 30 min at 4°C to remove the cell debris and cells and stored at −80°C. For urinary protein extraction, the urine samples were centrifuged at 12,000 × g for 30 min at 4°C again. Four milliliters of each sample was reduced with 20 mmol/L dithiothreitol (DTT, Sigma) at 95°C for 5 min and alkylated with 50 mmol/L iodoacetamide (IAA, Sigma) for 40 min in the dark. The urine samples were precipitated with six volumes of acetone at −20°C for 24 h and then centrifuged at 12,000 × g for 30 min at 4°C. Four milliliters of acetone were used to resuspend the pellets. After another centrifugation of 12,000 × g for 30 min, the pellets were dissolved in lysis buffer [8 mol/L urea, 2 mol/L thiourea, 50 mmol/L Tris, and 25 mmol/L dithiothreitol (DTT)]. Finally, the supernatants were quantified using the Bradford assay.

For trypsin digestion, 100 micrograms from each sample was digested with trypsin (Trypsin Gold, Mass Spec Grade, Promega, Fitchburg, WI, USA) using filter-aided sample preparation (FASP) methods (12). Briefly, the protein in each sample was loaded into a 10 kDa filter device (Pall, Port Washington, NY, USA). After washing two times with urea buffer (UA, 8 mol/L urea and 0.1 mol/L Tris-HCl) (pH 8.50) and three times with 25 mmol/L NH4HCO3 solutions, the samples were then digested with trypsin overnight at 37°C (enzyme-to-protein ratio of 1: 50). The digested peptides were desalted using Oasis HLB cartridges (Waters, Milford, MA, USA) and dried by vacuum evaporation (Thermo Fisher Scientific, Bremen, Germany). The digested peptides was dissolved in 0.1% formic acid and diluted to a concentration of 0.5 μg/μL and quantified by the BCA assay (23225, Thermo Fisher Scientific).

One microgram of peptides from each sample was loaded into a trap column (75 μm ^*^ 2 cm, 3 μm, C18, 100 Å) at a flow rate of 0.25 μL/min and then separated with a reversed-phase analytical column (75 μm ^*^ 250 mm, 2 μm, C18, 100 Å) with an EASY-nLC 1200 HPLC system (Thermo Fisher Scientific, USA). Peptides were eluted with a gradient of 4%-35% buffer B (0.1% formic acid in 80% acetonitrile) for 90 min and then analyzed with an Orbitrap Fusion Lumos Tribrid Mass Spectrometer (Thermo Fisher Scientific, Waltham, MA, USA). The MS data were acquired in high-sensitivity mode and at a spray voltage of 2.4 kV. A full MS scan was acquired within a 350–1,550 m/z range with the resolution set to 60,000. The MS/MS scan was acquired in Orbitrap mode with a resolution of 30,000. The cycle time was set to 3 s, and the HCD collision energy was set to 30%. Typical mass spectrometric conditions included the following: automatic gain control (AGC) targets of 4 × e5 ions for full scans and 5 × e4 for MS/MS scans; maximum injection time of 50 ms for full scans and 45 ms for MS/MS scans, and dynamic exclusion for 30 s.

### Data Analysis

Proteins were identified and quantified against the complete human proteins in the SwissProt database (20,227 sequences) using Proteome Discover 2.1 software (Thermo Fisher Scientific) with SEQUEST and Mascot search engines (version 2.5.1, Matrix Science, London, UK). The following parameters were set: trypsin digestion; missed cleavage sites ≤2; fixed modification = carbamido methylation of cysteines; variable modification = oxidation of methionine; peptide mass tolerance = 10 ppm; and fragment mass tolerance = 0.02 Da. A false discovery rate (FDR) of < 1% and at least two unique peptides were used to identify and quantify proteins.

Comparisons of the groups before and after treatment groups were conducted using two kinds of criteria: one-way ANOVAs or two-sided paired *t*-tests. Multiple comparisons were conducted using one-way ANOVA with Bonferroni's correction. The differential proteins were selected according to *P* < 0.05 and a fold change ≥1.5 or ≤0.67. All results are expressed as the mean ± standard deviation.

### Functional and Network Analysis

For getting more information associated treatment response, differential proteins without *p*-values' correction were annotated for functional analysis. We used the “Wu Kong” platform (https://www.omicsolution.org/wkomics/main/) for Orthogonal projection on latent structure-discriminant analysis (OPLS-DA) and enrichments of biological processes (BP), molecular functions (MF), and cellular components (CC) (based on Gene Ontology terms). The UniProt accession numbers of differentially expressed proteins were uploaded to ingenuity pathway analysis (IPA) software (QIAGEN, Redwood City, CA, USA) for molecular and cellular function and canonical pathway annotations. The proteins were mapped to available canonical pathways and ranked by *p*-values (http://inparanoid.sbc.su.se/cgi-bin/index.cgi) (13). The whole genome was selected as the background when performing the IPA analysis annotation. Functions of differential proteins were searched in reported studies based on the PubMed database (https://pubmed.ncbi.nlm.nih.gov). Protein interaction network analysis was performed using STRING software (https://string-db.org/cgi/input.pl) based on the STRING database (14). All of the raw data and mass spectrometry proteomics data have been deposited to the ProteomeXchange Consortium (http://proteomecentral.proteomexchange.org) via the iPROX partner repository with the dataset identifier PXD025608.

## Results

### Demographic and Clinical Characteristics of Patients in the First Trial

For patients who were diagnosed with depression, a 12-week treatment of escitalopram was applied, with the dose increasing from 5 mg/kg to 20 mg/kg once a day. Urine samples were collected at baseline, 2 weeks, and 12 weeks of treatment for the urinary proteomics analysis. The treatment efficacy was evaluated after 12 weeks of treatment. When the score reduction rate was >50%, the treatment was considered effective. The demographic and clinical characteristics of the subjects (baseline, W2 and W12 of treatment from nine respondent patients, *n* = 27) are presented in Supplemental Material 1.

### Urine Proteome Changes at the Early Stage of Escitalopram Treatment

Twenty-seven urine samples from nine respondents to escitalopram were selected to investigate the early changes in the urine proteome caused by escitalopram treatment. A total of 1,493 urinary proteins were identified. The two-sided paired *t*-test was conducted to screen differential proteins with the screening criteria fold change ≥1.5 or ≤0.67 and *p*-value < 0.05. Compared with the baseline (W0) before treatment, 16 and 25 differential proteins were identified at weeks 2 and 12, respectively (Supplemental Materials 2, 3). Protein alpha-1-antitrypsin was the overlapping differential protein at the two time points.

When the one-way ANOVA with Bonferroni's correction was performed, only seven differential proteins were identified differentially expressed with the same criteria (fold change ≥1.5 or ≤0.67 and *p*-value < 0.05). The result is shown in Table 1. Obviously, the screening criteria with multiple comparison and *p*-values' correction is more stringent and has a lower false positive rate. Nevertheless, the criteria without *p*-values' correction test is more relaxed with a higher false positive rate and a lower false negative rate, which provides more information about the changes of treatment response and is easier to identify the correlation with biological functions. Therefore, we provided both results of the two different screening criteria. As several recent researches indicated, the combination of several biomarkers reflecting changes in different biological mechanisms may be a promising direction for future research of MDD (15, 16). At the current initial stage of urinary biomarkers' researches, more comprehensive and sufficient clues that correlated with disease would be beneficial. Therefore, the less stringent differential urinary proteins were used for functional enrichment analysis and comparisons between pilot studies.

**Table 1:** Differential proteins from two trials after one-way ANOVA with *p*-values' correction.

**Group**	**Accession**	**Protein name**	**ANOVA-*p***	**Adiusted.*p*-w2**	**Adiusted.*p*-w12**	**FC-W2**	**FC-W12**	**Related to major depressive disorder**	**Related to psychiatric disorders or processes**
The first pilot-response group	COF1_HUMAN	Cofilin-1	3.36E-02	2.47E-02	/	1.72	/		Parkinson's disease (17)
	CD97_HUMAN	Adhesion G protein-coupled receptor E5	1.83E-02	1.21E-02	/	0.67	/		
	UPAR_HUMAN	Urokinase plasminogen activator surface receptor	4.54E-02	/	/	/	0.44		
	GLOD4_HUMAN	Glyoxalase domain-containing protein 4	4.50E-03	2.60E-03	/	2.31	/		
	CLIC1_HUMAN	Chloride intracellular channel protein 1	4.69E-02	2.86E-02	/	2.88	1.88		
	AQP2_HUMAN	Aquaporin-2	3.41E-02	/	/	/	0.65		
	CHM2A_HUMAN	Charged multivesicular body protein 2a	3.61E-02	/	3.88E-02	/	0.42		
The second pilot-response group	VP13D_HUMAN	Vacuolar protein sorting-associated protein 13D	8.00E-04	1.70E-03	1.70E-03	0.32	0.32		IL-7 signaling
	CAYP1_HUMAN	Calcyphosin	3.19E-02	2.16E-02	/	3.67	2.83		Neurogliomatosis (18)
	GMDS_HUMAN	GDP-mannose 4,6 dehydratase	1.56E-02	/	1.04E-02	/	0.60		Notch signaling
	ASXL1_HUMAN	Polycomb group protein ASXL1	3.30E-03	4.06E-02	2.00E-03	0.52	0.29		Neural Crest Development (19)
	TACC2_HUMAN	Transforming acidic coiled-coil-containing protein 2	4.92E-02	/	3.00E-02	0.63	0.25		Neural progenitor cells migration
	KREM1_HUMAN	Kremen protein 1	2.36E-02	/	1.47E-02	0.62	0.40		Regulate CNS patterning (20)
	FLNA_HUMAN	Filamin-A	2.28E-02	/	1.45E-02	0.66	0.49		Axon regeneration (21)
	AKAP9_HUMAN	A-kinase anchor protein 9	3.30E-02	/	2.24E-02	/	0.06		Synapse function
	DOPD_HUMAN	D-dopachrome decarboxylase	2.06E-02	/	4.09E-02	/	1.62	Pathogenic contribution to MDD (22)	
	ARPC4_HUMAN	Actin-related protein 2/3 complex subunit 4	4.95E-02	/	3.21E-02	0.67	0.47	Differentially expressed in MDD (23)	
The second pilot-non-response group	MEGF9_HUMAN	Multiple epidermal growth factor-like domains protein 9	2.00E-04	2.00E-04	0.0025	0.293	0.4483		CNS development (24)
	MRC2_HUMAN	C-type mannose receptor 2	2.90E-03	1.70E-03	/	0.554	/		
	GPC4_HUMAN	Glypican-4	2.49E-02	1.44E-02	/	0.573	/		CNS development
	T106B_HUMAN	Transmembrane protein 106B	1.80E-02	1.33E-02	/	0.385	/		Frontal-temporal dementia
	SFRP4_HUMAN	Secreted frizzled-related protein 4	2.50E-03	6.40E-03	0.0036	0.582	0.5522		PI3K/AKT signaling (25)
	IBP5_HUMAN	Insulin-like growth factor-binding protein 5	3.39E-02	/	0.0207	3.4	5		Neurogliomatosis (26)
	GALNS_HUMAN	N-acetylgalactosamine-6-sulfatase	8.50E-03	4.80E-03	/	0.613	/		
	BROX_HUMAN	BRO1 domain-containing protein BROX	2.62E-02	1.51E-02	/	0.571	/		
	GNA11_HUMAN	Guanine nucleotide-binding protein subunit alpha-11	1.54E-02	9.60E-03	/	0.5	0.6695		Hypothalamic neurons regulation
	ENPP4_HUMAN	Bis(5'-adenosyl)-triphosphatase ENPP4	3.21E-02	2.24E-02	/	0.105	0.3684		
	K1549_HUMAN	UPF0606 protein KIAA1549	3.64E-02	/	/	0.471	/		
	PSA1_HUMAN	Proteasome subunit alpha type-1	3.84E-02	/	/	0.484	/		Parkinson's disease (27)
	RAB5C_HUMAN	Ras-related protein Rab-5C	1.89E-02	3.35E-02	/	0.625	/		
	LRP3_HUMAN	Low-density lipoprotein receptor-related protein 3	3.48E-02	4.07E-02	/	0.385	/		Synapse remodeling (28)
	APLP2_HUMAN	Amyloid-like protein 2	3.05E-02	4.58E-02	/	0.645	/		Synaptic inhibition (29)
	MTPN_HUMAN	Myotrophin	4.45E-02	2.71E-02	/	4.667	3		Neuronal differentiation
	CDD_HUMAN	Cytidine deaminase	1.68E-02	1.57E-02	/	9	2.25		
	HXK1_HUMAN	Hexokinase-1	1.43E-02	1.56E-02	/	20	3		Neuroprotection against oxidative stress (30)
	MYLK_HUMAN	Myosin light chain kinase, smooth muscle	3.05E-02	1.86E-02	/	2.75	1.6875		Neurotransmitter release; Notch signaling
	FLNB_HUMAN	Filamin-B	2.18E-02	1.87E-02	/	1.93	/		Periventricular nodular ectopic with epileptic seizure (31)
	TFF1_HUMAN	Trefoil factor 1	3.20E-02	1.92E-02	/	1.661	/	GWAS analysis of MDD (32)	
	BMPR2_HUMAN	Bone morphogenetic protein receptor type-2	3.87E-02	4.19E-02	/	1.629	/		
	S12A1_HUMAN	Solute carrier family 12 member 1	4.89E-02	3.24E-02	/	0.558	/		
	RL11_HUMAN	60S ribosomal protein L11	1.37E-02	1.57E-02	/	∞	∞		
	RNF13_HUMAN	E3 ubiquitin-protein ligase RNF13	2.62E-02	/	0.0212	/	0.6216		
	NPNT_HUMAN	Nephronectin	9.80E-03	/	0.0054	/	0.5833		Integrin binding; p38 MAPK signaling (33)
	TCPB_HUMAN	T-complex protein 1 subunit beta	4.28E-02	/	0.0399	1.571	4.7143		Dopamine receptor function (34)
	H2B3B_HUMAN	Histone H2B type 3-B	2.75E-02	/	0.0158	1.63	2.2222		
	LAIR2_HUMAN	Leukocyte-associated immunoglobulin-like receptor 2	4.01E-02	/	0.0274	/	0.6042		
	PRIO_HUMAN	Major prion protein	1.70E-02	/	0.011	/	0.67	Protein markers of MDD and suicide (35)	Synaptic plasticity; GABA function (36)
	TFF2_HUMAN	Trefoil factor 2	1.42E-02	/	0.011	/	/		Maturation and migration of dendritic cells (37)
	H10_HUMAN	Histone H1.0	1.76E-02	/	0.0467	/	2.7778		
	H2B1A_HUMAN	Histone H2B type 1-A	4.22E-02	/	0.0297	/	1.7692		
	CHMP3_HUMAN	Charged multivesicular body protein 3	7.70E-03	/	0.0229	0.5	4		Alzheimer's disease (38)
	FGL2_HUMAN	Fibroleukin	7.20E-03	/	0.0172	/	2.2553		Neuroprotection (39)
	TICN1_HUMAN	Testican-1	3.79E-02	/	/	/	2		Alzheimer's disease (40); CNS development

*Urinary proteins previously reported associated with MDD or other psychiatric disorders and processes are annotated according to Pubmed or Uniprot database.

*“/” suggests the value cannot meet the screen criteria.

### Urine Differential Protein Function Analysis in the Early Stage of Escitalopram Treatment

**Figure 1:**
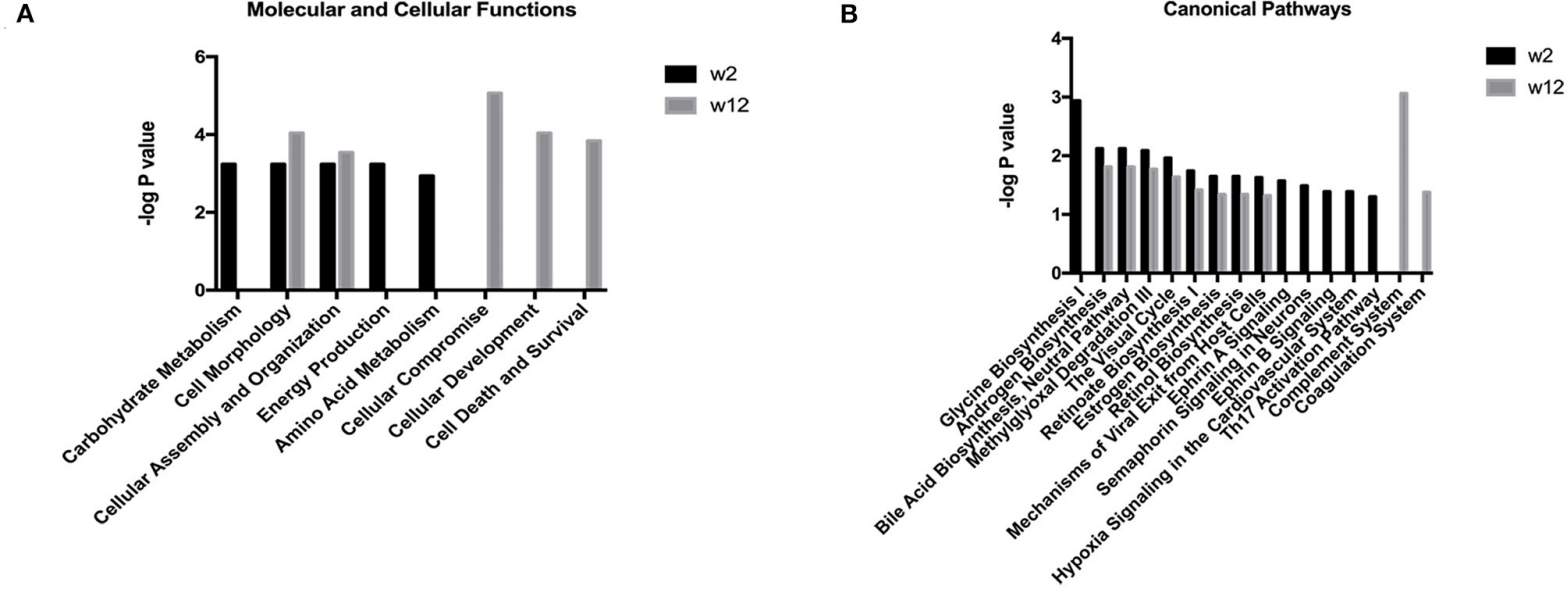
Functional analysis of differential proteins from the treatment respondent group in the first trial. Functional annotations including the molecular and cellular functions and canonical pathways were generated by ingenuity pathway analysis (IPA). The criteria of two-sides paired *t*-tests with *P* < 0.05 and fold change ≥1.5 or ≤0.67 were applied for screening differential proteins. **(A)** Molecular and cellular functions at week 2 and week 12 after escitalopram treatment. **(B)** Canonical pathway annotation at week 2 and week 12 after escitalopram treatment.

Functional enrichment analysis was performed for the differential proteins using the IPA database. The results are shown in Figure 1. The main molecular and cellular functions of the differential proteins were categorized in terms of cell morphology, cellular assembly and organization. After 2 weeks of treatment, the differential proteins were enriched in Ephrin A signaling, Ephrin B signaling, semaphorin signaling in neurons, oxidative stress and the Th17 activation pathway. After 12 weeks of treatment, the differential proteins were enriched in the complement system and the coagulation system. The pathways that were enriched together at the two time points were androgen biosynthesis, bile acid biosynthesis, methylglyoxal degradation and retinol biosynthesis.

### Demographic and Clinical Characteristics of Patients in the Second Trial

According to the first pilot study, our results suggested that urinary proteome changes associated with escitalopram treatment can be reflected at the very early stage of the therapy. Therefore, sixty urine samples from 10 respondents and 10 non-respondents to escitalopram (baseline, 2 weeks treatment and 12 weeks treatment) were collected for proteomic analysis in the second pilot study. These samples were selected to identify whether urine can distinguish varying degrees of escitalopram treatment response. Detailed demographic information is shown in Supplemental Material 4. Bonferroni's multiple comparisons test was performed to identify the consistency of demographic and clinical characteristics. The results (Supplemental Material 4) showed that there were no significant differences of demographic characteristics at the baseline and a significant difference in HAMD score after 12 weeks' treatment between the response and non-response group.

### Urine Proteome Changes of the Escitalopram Response Group and Non-response Group

After label-free LC-MS/MS quantitation, a total of 2,197 (week 2) and 2,326 (week 12) urinary proteins with at least two unique peptides were identified based on <1% FDR at the protein level. The screening criteria were a fold change ≥ 1.5 or ≤0.67 and a *p*-value < 0.05. When the one-way ANOVA with Bonferroni's correction was performed, 10 and 36 differential proteins were identified differentially expressed in the response and non-response group. The result is shown in Table 1. When the two-sides paired *t*-tests were performed, 20, 60, 25, and 59 differential proteins were identified in the response-w2, non-response-w2, response-w12 and non-response-w12 groups, respectively (Supplemental Materials 5–8).

After 2 weeks of treatment, 20 and 60 differential proteins were identified in the escitalopram response and non-response groups, respectively. In the response group, fourteen of 20 have been associated with the pathogenic and treatment processes of MDD, and 4 have been reported as biomarkers of MDD. Additionally, these 4 proteins had the same changing trend as the studies reported. In the non-response group, twenty-eight of 60 differential proteins have been associated with the pathogenic and treatment processes of MDD, and sepiapterin reductase has been reported as a biomarker of novel candidates for the study of antidepressant pharmacogenetics.

After 12 weeks of treatment, 25 and 59 differential proteins were identified in the response and non-response groups, respectively. In the response group, 13 of 25 have been associated with the pathogenic and treatment processes of MDD, and 6 have been reported as biomarkers of MDD. In the non-response group, 31 of 59 differential proteins were associated with the pathogenic and treatment processes of MDD, and eight were reported as biomarkers of MDD.

The overlap of these differential proteins in response W2, response W12, non-response W2 and non-response W12 is shown by a Venn diagram in Supplemental Material 9. In the response group, two common urinary differential proteins were identified, including alpha-1,6-mannosylglycoprotein 6-beta-N-acetylglucosaminyltransferase A and actin-related protein 2/3 complex subunit 4. In the non-response group, there were seven common differential proteins: ephrin type-A receptor 10, secreted frizzled-related protein 4, multiple epidermal growth factor-like domains protein 9, insulin-like growth factor-binding protein 5, ubiquitin-conjugating enzyme E2 variant 3, talin-1 and mucin-5B. The trend of the overlapping proteins was consistent between time points. In addition, the differential proteins were significantly separated at four different time points, indicating that the changes in urine proteins were different at different time points and varying degrees of escitalopram response. The Venn result showed that the urine proteome was highly sensitive.

### Functional Annotation of Differential Proteins Before and After Treatment

**Figure 2:**
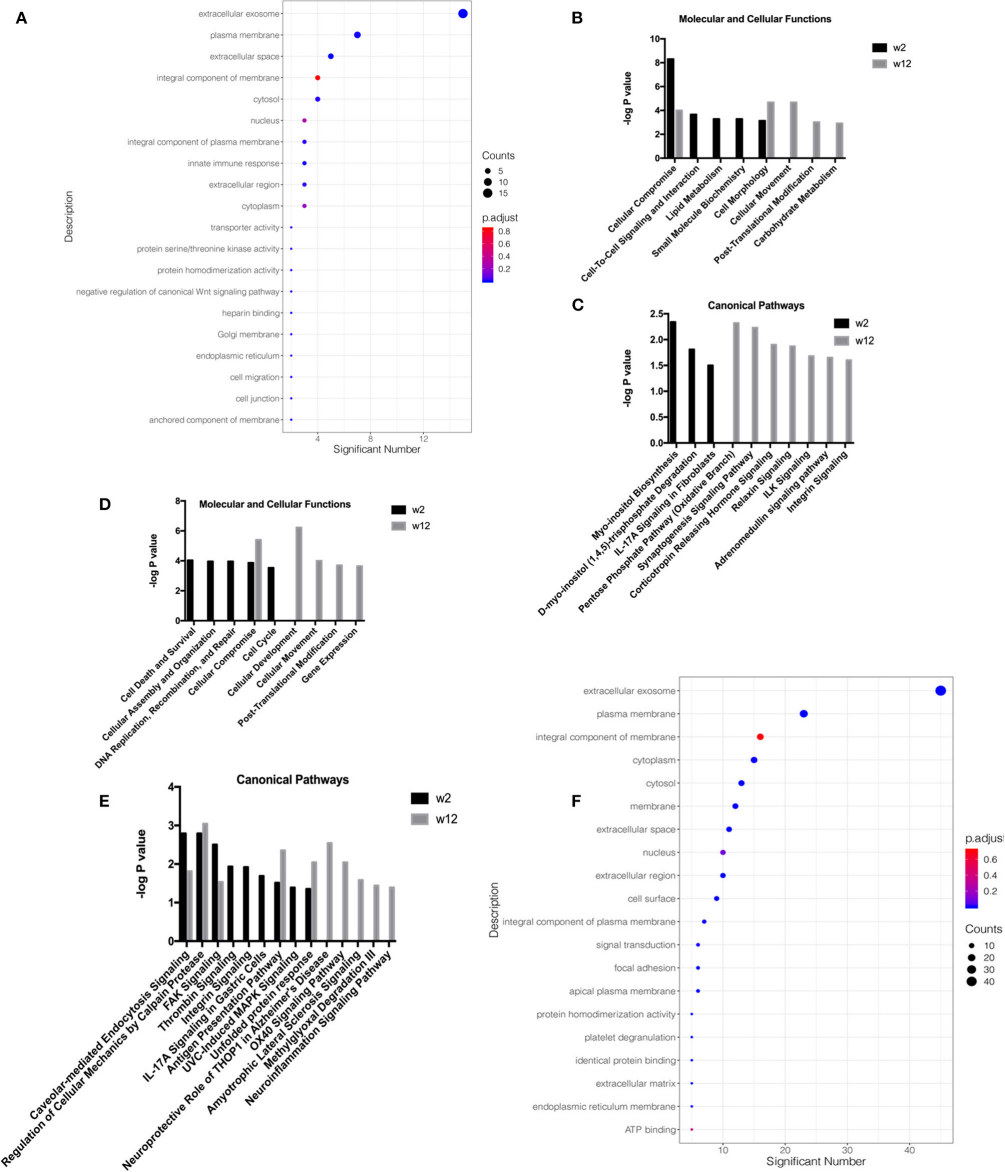
Functional analysis of differential proteins from the treatment response and non-response groups in the second trial. Functional annotations were generated by Gene Ontology (GO) and ingenuity pathway analysis (IPA). GO analysis of the differential proteins was performed to study the specific molecular functions (MF), biological processes (BP), and cellular components (CC) (the top 20 counts are shown, adjusted *P* < 0.05 with BH correction). IPA was performed to study the molecular and cellular functions and canonical pathway annotation. The criteria of two-sides paired *t*-tests with *P* < 0.05 and fold change ≥1.5 or ≤0.67 was applied for screening differential proteins. **(A)** GO analysis on week 2 in respondent group. **(B)** Molecular and cellular functions at week 2 and week 12 in the respondent group. **(C)** Canonical pathway annotation at week 2 and week 12 in the respondent group. **(D)** Molecular and cellular functions at week 2 and week 12 in the non-respondent group. **(E)** Canonical pathway annotation at week 2 and week 12 in the non-respondent group. **(F)** GO analysis on week 2 in non-respondent group.

Gene ontology (GO) analysis of the differential proteins was performed to study the specific molecular functions (MF), biological processes (BP), and cellular components (CC) (the top 20 counts are shown) (Figures 2A,D; Supplementary Materials 10A,B). Functional enrichment analyses based on GO revealed that differential proteins, including extracellular exosomes, ranked first at all time points and groups. Wnt signaling was enriched in the response-w2 group, and signal transduction and platelet degranulation were enriched in the non-response-w2 group.

To explore neurological disorders and investigate biological functions, the ingenuity pathway analysis (IPA) database was chosen to integrate and comprehensively analyze the quantitative information of differential proteins in the response-w2, response-w12, non-response-w2 and non-response-w12 groups. Ten representative statistically canonical pathways of the response-w2 and response-w12 groups in the ingenuity pathway analysis are shown in Figures 2B,C. Myo-inositol biosynthesis, D-myo-inositol (1, 4, 5)-trisphosphate degradation and IL-17A signaling in fibroblasts were overrepresented at the response-w2 time point. In the response-w12 group, the pentose phosphate (oxidative branch), synaptogenesis signaling, corticotropin releasing hormone signaling, relaxin signaling, ILK signaling, adrenomedullin signaling and integrin signaling pathways were enriched. Fourteen representative statistically canonical pathways of the non-response-w2 and non-response-w12 groups in the ingenuity pathway analysis are shown in Figures 2E,F. Caveolar-mediated endocytosis signaling, calpain protease, FAK signaling, antigen presentation and unfolded protein response pathways were enriched in the non-response-w2 and non-response-w12 groups. In addition, the thrombin signal, IL-17A signal and MAPK signal pathways were significantly changed in the non-response group at week 2. After 12 weeks of treatment, the neuroprotective effects of THOP1, the OX40 signaling pathway, the ALS signaling pathway, and the methylglyoxal and neuroinflammatory signaling pathways were significantly changed.

### Urine Differential Proteins Are Able to Distinguish Different Degrees of Escitalopram Response in the Second Group of Patients After 2 Weeks of Treatment

**Figure 3:**
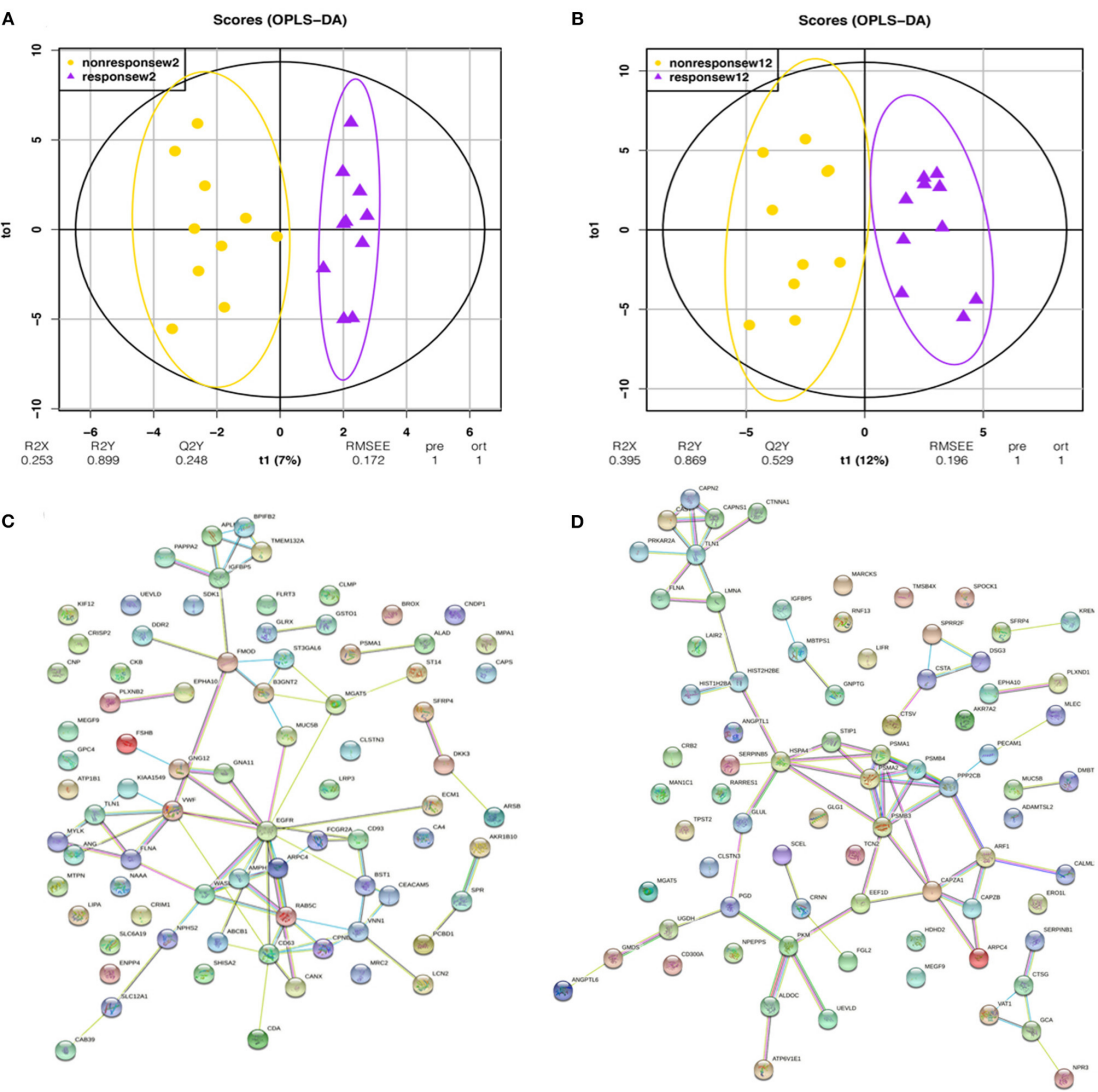
OPLS-DA and PPI analysis of urinary differential proteins in the second trial. OPLS-DA and PPI analyses were performed with the union differential proteins of respondent and non-respondent to escitalopram group. The criteria of two-sides paired *t*-tests with *P* < 0.05 and fold change ≥1.5 or ≤0.67 were applied for screening differential proteins. **(A)** OPLS-DA analysis shows an obvious discrimination between respondent and non-respondent patients after 2 weeks of treatment. **(B)** OPLS-DA analysis shows an obvious discrimination between respondent and non-respondent patients after 12 weeks of treatment. **(C)** Protein interaction network of urinary differential proteins after 2 weeks of treatment. **(D)** Protein interaction network of urinary differential proteins after 12 weeks of treatment.

Orthogonal projection on latent structure-discriminant analysis (OPLS-DA) and protein-protein interaction (PPI) network analysis were performed for the union differential proteins of the escitalopram response and non-response groups at week 2 or week 12 time point, and the results are shown in Figures 3A–D. OPLS-DA analysis showed a clear discrimination for response and non-response patients at both the 2 weeks' and 12 weeks' time points after escitalopram treatment. *R*^2^ (overall goodness of fit), Q^2^ (overall goodness of prediction) were examined. After 2 weeks' treatment, the model had an *R*^2^X value of 0.253, *R*^2^Y value of 0.899 and a Q^2^ value of 0.248. The model proved a possibility to discriminate different degrees of escitalopram. To predict precise treatment response, more samples are needed for training and verification. The PPI enrichment *p*-values were 1.14e-10 and 1.62e-06, which suggested a tight correlation among these proteins.

### Comparison of Differential Proteins in the Escitalopram Response Groups Between Two Trials

When comparing the differential proteins produced by during the two trials in escitalopram response patients, few overlapping differential proteins were observed (Supplemental Material 11). After 2 weeks of treatment, there were no overlapping differential proteins between the first and second trials of samples, and after 12 weeks of treatment, there were two overlapping differential proteins: synaptic vesicle membrane protein vat-1 homolog (involved in the regulation of oxidoreductase activity) and amiloride-sensitive amine oxidase [copper-containing] (reported to be related to Alzheimer's disease and involved in the regulation of nitric oxide pathway). A comparison of biological pathways showed that the IL-17 signaling, glycine and heme biosynthesis, and histamine degradation pathways overlapped and were enriched together.

## Discussion

In the present study, the urine proteome changes of escitalopram treatment at 2 weeks' and 12 weeks' time points were identified using liquid chromatography coupled with tandem mass spectrometry (LC-MS/MS). Intriguingly, a significant number of identified differential proteins and enriched pathways were found to be associated with MDD or related pathways, which support the correlation between the urinary proteome changes and treatment response of MDD patients.

In the first pilot study, six of 16 differential proteins after 2 weeks' treatment have been annotated to be associated with MDD or psychiatric processes. In the second pilot study, 14 of 20 and 28 of 60 differential proteins of response and non-response groups after 2 weeks' treatment have been annotated to be associated with MDD or psychiatric processes. All of the annotated details of each time points are presented in Supplemental Materials. In addition, several enriched biological processes and pathways reveal a similar correlation with depression and antidepressant drug's treatment. For example: (1). Oxidative stress (enriched by HSP90 and DDAH1): Oxidative stress refers to the biologically damaging effects of free radicals, there is evidence suggesting that oxidative stress may be increased in a number of psychiatric disorders, including depression (41). Increased concentrations of NO enhance the production of reactive nitrogen species (RNS) and reactive oxygen species (ROS), which are associated with an increase in pro-inflammatory cytokines. Several recent studies have implicated nitric oxide (NO) as a critical regulator of neuroinflammation, thus suggesting a possible role in the pathophysiology of MDD (42). (2). MAPK signaling (enriched in both two trials): P38 mitogen-activated protein kinase (MAPK) is a crucial target for chronic inflammatory diseases. P38 MAPK is a class of MAPKs responsive to stress stimuli such as inflammatory cytokines and reactive oxygen species (ROS) and participates in potential mechanisms of depression (43). (3). Notch signaling and PI3K-Akt signaling: Notch1 was reported to regulate the PI3K-AKT-mTOR1 signaling (44) and regulates motor neuron differentiation. The PI3K-Akt pathway is one of the classical cell cycle regulation pathways, and is crucial to promoting neuronal survival and neurogenesis (45). It is also a pathway that is effectively targeted by many anti-depressants (46). (4). interleukin-17 signaling pathway (enriched in both two trials): has been reported to be involved in depression development (47). These findings offer a novel possible way that using urine proteome changes to predict treatment response of MDD timely during antidepressant medication.

Major depressive disorder (MDD) is a severe psychiatric disorder. However, due to the strong heterogeneity and complex pathogenesis of depression, the research results and clinical application of biomarkers remains unsatisfactory. In this study, a before and after self-control study was designed to avoid the heterogeneity among individuals. Our first pilot study showed that urine proteomic changes can reflect the escitalopram treatment response even at the very early time (i.e., week 2). And we further showed the possibility that urinary proteome changes can distinguish the response and non-response group in the second trial. In addition, the differential proteins were correlated with the pathological mechanisms or antidepressant treatment of the MDD disease. These findings provide clues for predicting efficient treatment measures in MDD patients and may shorten the time for evaluating clinical medication during the rational selection of next step treatments.

In the current initial phase, we are trying to explore and provide valuable clues for further investigation of escitalopram treatment response biomarkers. The differential proteins identified in this study may not cover all the urinary proteomic changes from MDD patients with escitalopram treatment. The candidate treatment response biomarkers identified in each pilot study may only be applicable to patients with specific subtypes. Our preliminary findings proposed a novel perspective to identify early treatment response biomarkers of antidepressants through urinary proteomics and suggested that additional trials and resources would be needed to further develop this area. More attention and resources would be beneficial to develop urinary biomarkers.

## Data Availability Statement

The datasets presented in this study can be found in online repositories. The names of the repository/repositories and accession number(s) can be found at: http://www.proteomexchange.org/~PXD025608.

## Ethics Statement

The studies involving human participants were reviewed and approved by Human Research and Ethics Committee of Beijing Anding Hospital (#2017-24). The patients/participants provided their written informed consent to participate in this study.

## Author Contributions

YH, JY, and YG contributed to conception and design of the study. JZ and ML organized the database. JW performed the statistical analysis and wrote sections of the manuscript. YH wrote the first draft of the manuscript. All authors contributed to manuscript revision, read, and approved the submitted version.

## Funding

This research was supported by the National Key Research and Development Program of China (2018YFC0910202 and 2016YFC1306300), the Beijing Natural Science Foundation (7173264 and 7172076), the Beijing Cooperative Construction Project (110651103), the Beijing Normal University (11100704), the Peking Union Medical College Hospital (2016–2.27), and Beijing Biobank of Clinical Resources–Mental Disorders, BBCR-MD.

## Conflict of Interest

The authors declare that the research was conducted in the absence of any commercial or financial relationships that could be construed as a potential conflict of interest.

## Publisher's Note

All claims expressed in this article are solely those of the authors and do not necessarily represent those of their affiliated organizations, or those of the publisher, the editors and the reviewers. Any product that may be evaluated in this article, or claim that may be made by its manufacturer, is not guaranteed or endorsed by the publisher.

## References

[1] Bromet E Andrade LH Hwang I Sampson NA Alonso J de Girolamo G. Cross-national epidemiology of DSM-IV major depressive episode. BMC Med. (2011) 9:90. 10.1186/1741-7015-9-90 21791035 PMC3163615

[2] Depression and Other Common Mental Disorders: Global Health Estimates. Geneva: World Health Organization (2017).

[3] Lecrubier Y. Widespread underrecognition and undertreatment of anxiety and mood disorders: results from 3 European studies. J Clin Psychiatry. (2007) 68(Suppl. 2):36–41. 10.1109/TIE.2002.803172 17288506

[4] An M Gao Y. Urinary biomarkers of brain diseases. Genomics Proteomics Bioinformatics. (2015) 13:345–54. 10.1016/j.gpb.2015.08.005 26751805 PMC4747650

[5] Anderson IM Nutt DJ Deakin JF. Evidence-based guidelines for treating depressive disorders with antidepressants: a revision of the 1993 British Association for Psychopharmacology guidelines. British Association for Psychopharmacology. J Psychopharmacol. (2000) 14:3–20. 10.1177/026988110001400101 10757248

[6] Barbui C Hotopf M Amitriptyline V. The rest: still the leading antidepressant after 40 years of randomised controlled trials. Br J Psychiatry. (2001) 178:129–44. 10.1192/bjp.178.2.129 11157426

[7] Owens MJ Knight DL Nemeroff CB. Second-generation SSRIs: human monoamine transporter binding profile of escitalopram and R-fluoxetine. Biol Psychiatry. (2001) 50:345–50. 10.1016/S0006-3223(01)01145-3 11543737

[8] Pehrson AL Sanchez C. Serotonergic modulation of glutamate neurotransmission as a strategy for treating depression and cognitive dysfunction. CNS Spectr. (2014) 19:121–33. 10.1017/S1092852913000540 23903233 PMC3968911

[9] Celada P Bortolozzi A Artigas F. Serotonin 5-HT1A receptors as targets for agents to treat psychiatric disorders: rationale and current status of research. CNS Drugs. (2013) 27:703–16. 10.1007/s40263-013-0071-0 23757185

[10] Rush AJ Trivedi MH Ibrahim HM Carmody TJ Arnow B Klein DN. The 16-Item Quick Inventory of Depressive Symptomatology (QIDS), clinician rating (QIDS-C), and self-report (QIDS-SR): a psychometric evaluation in patients with chronic major depression. Biol Psychiatry. (2003) 54:573–83. 10.1016/S0006-3223(02)01866-8 12946886

[11] Kroenke K Spitzer RL Williams JB. The PHQ-9: validity of a brief depression severity measure. J Gen Intern Med. (2001) 16:606–13. 10.1046/j.1525-1497.2001.016009606.x 11556941 PMC1495268

[12] Wisniewski JR Zougman A Nagaraj N Mann M. Universal sample preparation method for proteome analysis. Nat Methods. (2009) 6:359–62. 10.1038/nmeth.1322 19377485

[13] Sonnhammer EL Ostlund G. InParanoid 8: orthology analysis between 273 proteomes, mostly eukaryotic. Nucleic Acids Res. (2015) 43:D234–9. 10.1093/nar/gku1203 25429972 PMC4383983

[14] Szklarczyk D Gable AL Lyon D Junge A Wyder S Huerta-Cepas J. STRING v11: protein-protein association networks with increased coverage, supporting functional discovery in genome-wide experimental datasets. Nucleic Acids Res. (2019) 47:D607–13. 10.1093/nar/gky1131 30476243 PMC6323986

[15] Shi Y Song R Wang L Qi Y Zhang H Zhu J. Identifying Plasma Biomarkers with high specificity for major depressive disorder: a multi-level proteomics study. J Affect Disord. (2020) 277:620–30. 10.1016/j.jad.2020.08.078 32905914

[16] Jentsch MC Van Buel EM Bosker FJ Gladkevich AV Klein HC Oude Voshaar RC. Biomarker approaches in major depressive disorder evaluated in the context of current hypotheses. Biomark Med. (2015) 9:277–97. 10.2217/bmm.14.114 25731213

[17] Oliveira da Silva MI Liz MA. Linking alpha-synuclein to the actin cytoskeleton: consequences to neuronal function. Front Cell Dev Biol. (2020) 8:787. 10.3389/fcell.2020.00787 32903460 PMC7434970

[18] Zhu Z Wang J Tan J Yao Y He Z Xie X. Calcyphosine promotes the proliferation of glioma cells and serves as a potential therapeutic target. J Pathol. (2021). 10.1002/path.5776. [Epub ahead of print]. 34370292 PMC9291001

[19] Matheus F Rusha E Rehimi R Molitor L Pertek A Modic M. Pathological ASXL1 mutations and protein variants impair neural crest development. Stem Cell Reports. (2019) 12:861–8. 10.1016/j.stemcr.2019.03.006 31006630 PMC6524927

[20] Davidson G Mao B del Barco Barrantes I Niehrs C. Kremen proteins interact with Dickkopf1 to regulate anteroposterior CNS patterning. Development. (2002) 129:5587–96. 10.1242/dev.00154 12421700

[21] Cho Y Park D Cavalli V. Filamin A is required in injured axons for HDAC5 activity and axon regeneration. J Biol Chem. (2015) 290:22759–70. 10.1074/jbc.M115.638445 26157139 PMC4566247

[22] Petralia MC Mazzon E Fagone P Basile MS Lenzo V Quattropani MC. Pathogenic contribution of the Macrophage migration inhibitory factor family to major depressive disorder and emerging tailored therapeutic approaches. J Affect Disord. (2020) 263:15–24. 10.1016/j.jad.2019.11.127 31818772

[23] Choi JE Lee JJ Kang W Kim HJ Cho JH Han PL. Proteomic analysis of hippocampus in a mouse model of depression reveals neuroprotective function of ubiquitin c-terminal hydrolase L1 (UCH-L1) via stress-induced cysteine oxidative modifications. Mol Cell Proteomics. (2018) 17:1803–23. 10.1074/mcp.RA118.000835 29959188 PMC6126396

[24] Brandt-Bohne U Keene DR White FA Koch M. MEGF9: a novel transmembrane protein with a strong and developmentally regulated expression in the nervous system. Biochem J. (2007) 401:447–57. 10.1042/BJ20060691 16981854 PMC1820795

[25] Hossein G Khanmohammadi M Sahranavard Fard P Heidarian Y Kazemnejad S Akhondi MM. Exogenous secreted frizzled-related protein-4 modulates steroidogenesis of rat granulosa cells through Wnt/beta-catenin and PI3K/AKT signaling pathways. Avicenna J Med Biotechnol. (2016) 8:159–68. 27920883 PMC5124252

[26] Cesi V Vitali R Tanno B Giuffrida ML Sesti F Mancini C. Insulin-like growth factor binding protein 5: contribution to growth and differentiation of neuroblastoma cells. Ann N Y Acad Sci USA. (2004) 1028:59–68. 10.1196/annals.1322.007 15650232

[27] Wang Y Wang Z. An integrated network analysis of mRNA and gene expression profiles in Parkinson's disease. Med Sci Monit. (2020) 26:e920846. 10.12659/MSM.920846 32210219 PMC7115122

[28] O'Sullivan NC McGettigan PA Sheridan GK Pickering M Conboy L O'Connor JJ. Temporal change in gene expression in the rat dentate gyrus following passive avoidance learning. J Neurochem. (2007) 101:1085–98. 10.1111/j.1471-4159.2006.04418.x 17298388

[29] Trillaud-Doppia E Paradis-Isler N Boehm J. A single amino acid difference between the intracellular domains of amyloid precursor protein and amyloid-like precursor protein 2 enables induction of synaptic depression and block of long-term potentiation. Neurobiol Dis. (2016) 91:94–104. 10.1016/j.nbd.2016.02.016 26921470

[30] Duarte AI Santos P Oliveira CR Santos MS Rego AC. Insulin neuroprotection against oxidative stress is mediated by Akt and GSK-3beta signaling pathways and changes in protein expression. Biochim Biophys Acta. (2008) 1783:994–1002. 10.1016/j.bbamcr.2008.02.016 18348871

[31] Cao M Liu C Wei Z Qiao X Deng Y. [New variants in FLNA gene cause periventricular nodular heterotopia and epileptic seizure in three cases]. Zhonghua Yi Xue Yi Chuan Xue Za Zhi. (2021) 38:626–30. 10.3760/cma.j.cn511374-20200312-00157 34247364

[32] Cheng S Guan F Ma M Zhang L Cheng B Qi X. An atlas of genetic correlations between psychiatric disorders and human blood plasma proteome. Eur Psychiatry. (2020) 63:e17. 10.1192/j.eurpsy.2019.6 32093803 PMC7315878

[33] Toraskar J Magnussen SN Chawla K Svineng G Steigedal TS. Nephronectin mediates p38 MAPK-induced cell viability via its integrin-binding enhancer motif. FEBS Open Bio. (2018) 8:1992–2001. 10.1002/2211-5463.12544 30524949 PMC6275265

[34] Mansuri MS Peng G Wilson RS Lam TT Zhao H Williams KR. Differential protein expression in striatal D1- and D2-dopamine receptor-expressing medium spiny neurons. Proteomes. (2020) 8:27. 10.3390/proteomes8040027 33066078 PMC7709116

[35] Dean B Tsatsanis A Lam LQ Scarr E Duce JA. Changes in cortical protein markers of iron transport with gender, major depressive disorder and suicide. World J Biol Psychiatry. (2020) 21:119–26. 10.1080/15622975.2018.1555377 30513246

[36] Kishimoto Y Hirono M Atarashi R Sakaguchi S Yoshioka T Katamine S. Impairment of cerebellar long-term depression and GABAergic transmission in prion protein deficient mice ectopically expressing PrPLP/Dpl. Sci Rep. (2020) 10:15900. 10.1038/s41598-020-72753-6 32985542 PMC7522223

[37] Sung GH Chang H Lee JY Song SY Kim HS. Pancreatic-cancer-cell-derived trefoil factor 2 impairs maturation and migration of human monocyte-derived dendritic cells *in vitro*. Anim Cells Syst (Seoul). (2018) 22:368–81. 10.1080/19768354.2018.1527721 30533259 PMC6282439

[38] Florentinus-Mefailoski A Bowden P Scheltens P Killestein J Teunissen C Marshall JG. The plasma peptides of Alzheimer's disease. Clin Proteomics. (2021) 18:17. 10.1186/s12014-021-09320-2 34182925 PMC8240224

[39] Jin SJ Liu Y Deng SH Liao LH Lin TL Ning Q. Neuroprotective effects of activated protein C on intrauterine inflammation-induced neonatal white matter injury are associated with the downregulation of fibrinogen-like protein 2/fibroleukin prothrombinase and the inhibition of pro-inflammatory cytokine expression. Int J Mol Med. (2015) 35:1199–212. 10.3892/ijmm.2015.2136 25777531 PMC4380123

[40] Barrera-Ocampo A Arlt S Matschke J Hartmann U Puig B Ferrer I. Amyloid-beta precursor protein modulates the sorting of testican-1 and contributes to its accumulation in brain tissue and cerebrospinal fluid from patients with Alzheimer disease. J Neuropathol Exp Neurol. (2016) 75:903–16. 10.1093/jnen/nlw065 27486134 PMC5015660

[41] Black CN Bot M Scheffer PG Cuijpers P Penninx BW. Is depression associated with increased oxidative stress? a systematic review and meta-analysis. Psychoneuroendocrinology. (2015) 51:164–75. 10.1016/j.psyneuen.2014.09.025 25462890

[42] Kudlow P Cha DS Carvalho AF McIntyre RS. Nitric oxide and major depressive disorder: pathophysiology and treatment implications. Curr Mol Med. (2016) 16:206–15. 10.2174/1566524016666160126144722 26812915

[43] Xie Z Huang S Xie S Zhou W Li C Xing Z. Potential correlation between depression-like behavior and the mitogen-activated protein kinase pathway in the rat hippocampus following spinal cord injury. World Neurosurg. (2021). 10.1016/j.wneu.2021.06.093. [Epub ahead of print]. 34271150

[44] Hales EC Taub JW Matherly LH. New insights into Notch1 regulation of the PI3K-AKT-mTOR1 signaling axis: targeted therapy of gamma-secretase inhibitor resistant T-cell acute lymphoblastic leukemia. Cell Signal. (2014) 26:149–61. 10.1016/j.cellsig.2013.09.021 24140475

[45] Manning BD Toker A. AKT/PKB signaling: navigating the network. Cell. (2017) 169:381–405. 10.1016/j.cell.2017.04.001 28431241 PMC5546324

[46] Pazini FL Cunha MP Rosa JM Colla AR Lieberknecht V Oliveira A. Creatine, similar to ketamine, counteracts depressive-like behavior induced by corticosterone via PI3K/Akt/mTOR pathway. Mol Neurobiol. (2016) 53:6818–34. 10.1007/s12035-015-9580-9 26660117

[47] Zeng NX Li HZ Wang HZ Liu KG Gong XY Luo WL. Exploration of the mechanism by which icariin modulates hippocampal neurogenesis in a rat model of depression. Neural Regen Res. (2022) 17:632–42. 10.4103/1673-5374.320993 34380904 PMC8504392

