# Supplementary material for: Label-Free Liquid Chromatography–Mass Spectrometry Proteomic Analysis of the Urinary Proteome for Measuring the Escitalopram Treatment Response From Major Depressive Disorder
Source: Front Psychiatry. 2021 Sep 30;12:700149. doi: 10.3389/fpsyt.2021.700149 (PMC8514635; doi:10.3389/fpsyt.2021.700149)
Supplement: Supplementary file 1 [file Data_Sheet_1.DOCX]

**Supplemental Material Legends**

**Supplemental material 1. Demographic and clinical characteristics of patients in the first trial.**

**Supplemental material 2. Differential proteins identified on week 2 in respondent group in the first trial.**

**Supplemental material 3. Differential proteins identified at week 12 in the respondent group in the first trial.**

**Supplemental material 4. Demographic and clinical characteristics of patients in the second trial.**

**Supplemental material 5. Differential proteins identified on week 2 in respondent group in the second trial.**

**Supplemental material 6. Differential proteins identified on week 2 in nonrespondent group in the second trial.**

**Supplemental material 7. Differential proteins identified at week 12 in the respondent group in the second trial.**

**Supplemental material 8. Differential proteins identified at week 12 in the nonrespondent group in the second trial.**

**Supplemental material 9. Overlap evaluation of the differential proteins identified at different time points and groups in the second trial.** (The criteria of two-sides paired t-tests with P < 0.05 and fold change ≥ 1.5 or ≤ 0.67 was applied).

**Supplemental material 10. Gene ontology (GO) analysis of urinary differential proteins at week 12 in the second trial.** GO analysis of the differential proteins was performed to study the specific molecular functions (MF), biological processes (BP), and cellular components (CC) (the top 20 counts are shown, adjusted p < 0.05 with BH correction). The criteria of two-sides paired t-tests with P < 0.05 and a fold change ≥ 1.5 or ≤ 0.67 was applied for screening differential proteins. (A) GO analysis on week 12 in respondent group. (B) GO analysis on week 12 in nonrespondent group.

**Supplemental material 11. Venn diagram of differential proteins at different time points in escitalopram respondent groups between two trials.** (The criteria of two-sides paired t-tests with P < 0.05 and fold change ≥ 1.5 or ≤ 0.67 was applied).

**Supplemental material 1 Demographic and clinical characteristics of patients in the first trial**

| Variables | baseline-R | R-w2 | R-w4 | R-w8 | R-w12 |
| --- | --- | --- | --- | --- | --- |
| Female/Male | 5/4 |  |  |  |  |
| Age(years) | 34.33±4.69 |  |  |  |  |
| BMI | 22.77±2.98 |  |  |  |  |
| HAMD scores | 21.78±6.16 |  | 7.33±4.24 | 5.33±3.08 | 3.22±2.28 |
| QIDS | 13.44±1.88 | 9.89±3.06 | 6.89±2.37 | 6.22±2.54 | 4.11±1.45 |
| YMRS | 0.56±0.88 |  | 0.44±1.01 | 0.33±0.71 | 0.44±1.01 |
| PHQ9 | 14.78±3.63 |  | 7.00±3.67 | 5.56±3.97 | 3.00±2.06 |

(R-Respondent，NR-Nonrespondent)

**Supplemental material 2 Differential proteins identified on week 2 in respondent group in the first trial**

| Accession | Protein name | Trend | FC | P | related to major depressive disorder | related to psychiatric disorders or processes | |
| --- | --- | --- | --- | --- | --- | --- | --- |
| P07900 | Heat shock protein HSP 90-alpha | ↑ | 2.27 | 2.11E-02 |  | psychiatric disease | |
| P34896 | Serine hydroxymethyltransferase, cytosolic | ↑ | 1.84 | 2.81E-02 |  |  | |
| P23528 | Cofilin-1 | ↑ | 1.72 | 1.36E-02 |  | Parkinson’s disease | |
| P06331 | Immunoglobulin heavy variable 4-34 | ↓ | 0.63 | 2.34E-02 |  |  |  |
| O94760 | N(G),N(G)-dimethylarginine dimethylaminohydrolase 1 | ↑ | 2.30 | 3.47E-02 | depressive-like behaviors |  | |
| P48960 | Adhesion G protein-coupled receptor E5 | ↓ | 0.67 | 1.17E-03 |  |  |  |
| P02511 | Alpha-crystallin B chain | ↑ | 2.27 | 3.08E-02 |  | Alexander disease | |
| Q969E1 | Liver-expressed antimicrobial peptide 2 | ↓ | 0.52 | 4.36E-02 |  |  |  |
| Q9HC38 | Glyoxalase domain-containing protein 4 | ↑ | 2.31 | 8.00E-03 |  |  |  |
| Q96C23 | Aldose 1-epimerase | ↑ | 2.00 | 4.62E-02 |  |  |  |
| P42330 | Aldo-keto reductase family 1 member C3 | ↑ | 2.40 | 1.12E-02 |  | Bipolar Disorder | |
| O00299 | Chloride intracellular channel protein 1 | ↑ | 2.88 | 1.33E-02 |  |  |  |
| Q15847 | Adipogenesis regulatory factor | ↑ | 1.77 | 2.12E-02 |  |  |  |
| P13693 | Translationally-controlled tumor protein | ↑ | 2.33 | 4.97E-02 | antidepressive drugs |  | |
| Q9UN37 | Vacuolar protein sorting-associated protein 4A | ↑ | 1.73 | 1.22E-02 |  |  |  |
| Q96P63 | Serpin B12 | ↓ | 0.15 | 4.70E-02 |  |  |  |

**Supplemental material 3 Differential proteins identified at week 12 in the respondent group in the first trial**

| Accession | Protein name | Trend | FC | P | Related to MDD | Related to psychiatric disorders or processes |
| --- | --- | --- | --- | --- | --- | --- |
| P19801 | Amiloride-sensitive amine oxidase [copper-containing] | ↓ | 0.54 | 1.65E-02 |  | Alzheimer's disease |
| P50995 | Annexin A11 | ↓ | 0.59 | 3.95E-02 |  |  |
| Q08345 | Epithelial discoidin domain-containing receptor 1 | ↓ | 0.62 | 9.05E-03 |  | Neuroticism |
| Q14393 | Growth arrest-specific protein 6 | ↓ | 0.60 | 3.30E-03 |  |  |
| Q15485 | Ficolin-2 | ↓ | 0.63 | 1.03E-02 |  | Accurately ischemic stroke (AIS) |
| Q8WVQ1 | Soluble calcium-activated nucleotidase 1 | ↓ | 0.61 | 2.51E-02 |  |  |
| P78380 | Oxidized low-density lipoprotein receptor 1 | ↑ | 1.82 | 2.32E-02 |  |  |
| P08133 | Annexin A6 | ↑ | 1.96 | 3.02E-02 |  | PI3K/AKT/mTOR signaling |
| P12277 | Creatine kinase B-type | ↓ | 0.41 | 3.92E-02 |  | Alzheimer's disease |
| P08754 | Guanine nucleotide-binding protein G(k) subunit alpha | ↓ | 0.51 | 3.37E-02 |  | Parkinson's disease |
| P02748 | Complement component C9 | ↓ | 0.28 | 3.22E-02 |  |  |
| Q9H444 | Charged multivesicular body protein 4b | ↓ | 0.59 | 3.07E-02 |  | Necrosis of astrocytes |
| Q8NFT8 | Delta and Notch-like epidermal growth factor-related receptor | ↓ | 0.61 | 2.49E-02 |  | Development of central nervous system |
| Q8TF66 | Leucine-rich repeat-containing protein 15 | ↓ | 0.50 | 1.71E-02 |  |  |
| P04732 | Metallothionein-1E | ↑ | 1.59 | 3.92E-02 |  |  |
| Q16706 | Alpha-mannosidase 2 | ↓ | 0.43 | 4.74E-02 |  | Neurogenesis |
| Q03405 | Urokinase plasminogen activator surface receptor | ↓ | 0.44 | 1.71E-02 |  |  |
| Q9H9H4 | Vacuolar protein sorting-associated protein 37B | ↓ | 0.50 | 9.21E-03 |  | Alzheimer's disease |
| P07307 | Asialoglycoprotein receptor 2 | ↓ | 0.46 | 1.03E-02 |  |  |
| P41217 | OX-2 membrane glycoprotein | ↓ | 0.53 | 1.33E-02 |  | CREB signaling; Neuroinflammation |
| O14594 | Neurocan core protein | ↓ | 0.63 | 3.16E-02 |  | Neuroblastoma |
| Q99536 | Synaptic vesicle membrane protein VAT-1 homolog | ↑ | 2.88 | 2.82E-02 |  |  |
| Q8NC42 | E3 ubiquitin-protein ligase RNF149 | ↑ | 2.18 | 3.75E-02 |  | MAPK signaling |
| P42330 | Aldo-keto reductase family 1 member C3 | ↑ | 2.10 | 4.70E-02 | Target of SSRIs |  |
| Q16581 | C3a anaphylatoxin chemotactic receptor | ↓ | 0.50 | 2.78E-02 |  | Degeneration of the optic nerve |

**Supplemental material 4 Demographic and clinical characteristics of patients in the second trial**

| Variables | baseline-R | R & NR Adjusted P Value (baseline) | R-w2 | R-w4 | R-w12 | R & NR  Adjusted P Value  (treatment) |
| --- | --- | --- | --- | --- | --- | --- |
| Female/Male | 5/5 |  |  |  |  |  |
| Age(years) | 27.82±4.47 | >0.99 |  |  |  |  |
| BMI | 22.84±2.89 | >0.99 |  |  |  |  |
| HAMD scores | 24.2±4.71 | 0.76 |  | 17±7.26 | 6.6±3.84 | 0.001 |
| QIDS | 16.1±4.65 | >0.99 | 13.5±4.60 | 9.8±3.49 | 6±3.23 | >0.99 |
| YMRS | 1±1.49 | >0.99 |  | 0.9±1.91 | 1.1±1.45 | >0.99 |
| PHQ9 | 17.8±4.98 | >0.99 |  | 11.9±4.23 | 6.4±3.17 | 0.48 |
| Variables | baseline-NR |  | NR-w2 | NR-w4 | NR-w12 |  |
| Female/Male | 5/5 |  |  |  |  |  |
| Age(years) | 28.64±8.03 |  |  |  |  |  |
| BMI | 22.34±2.95 |  |  |  |  |  |
| HAMD scores | 21.2±3.58 |  |  | 15.67±3.94 | 15.2±4.29 |  |
| QIDS | 15.8±3.46 |  | 11.3±3.68 | 10.33±3.04 | 8.3±2.75 |  |
| YMRS | 1.7±1.83 |  |  | 0.89±0.78 | 1.1±1.29 |  |
| PHQ9 | 16.6±4.81 |  |  | 12.78±6.69 | 10.1±4.53 |  |

(R-Respondent，NR-Nonrespondent)

**Supplemental material 5 Differential proteins identified on week 2 in respondent group in the second trial**

| Accession | Protein name | Trend | FC | P value | related to major depressive disorder | related to psychiatric disorders or processes | |
| --- | --- | --- | --- | --- | --- | --- | --- |
| P13716 | Delta-aminolevulinic acid dehydratase | ↓ | 0.62 | 4.44E-03 |  |  | |
| Q09328 | Alpha-1,6-mannosylglycoprotein 6-beta-N-acetylglucosaminyltransferase A | ↓ | 0.42 | 1.33E-02 |  | Schizophrenia | |
| Q9NP85 | Podocin | ↑ | 2.06 | 1.38E-02 |  |  | |
| P80188 | Neutrophil gelatinase-associated lipocalin | ↓ | 0.63 | 1.45E-02 | increased in MDD |  | |
| P08962 | CD63 antigen | ↓ | 0.46 | 1.83E-02 | involved in the platelet activation process, increased in MDD |  | |
| Q96FN5 | Kinesin-like protein KIF12 | ↑ | 3.22 | 2.08E-02 |  |  | |
| Q9UBP4 | Dickkopf-related protein 3 | ↓ | 0.50 | 2.12E-02 |  | Alzheimer’s Disease^[19]^ | |
| P29218 | Inositol monophosphatase 1 | ↓ | 0.20 | 2.58E-02 |  | Bipolar Disorder | |
| Q9Y376 | Calcium-binding protein 39 | ↓ | 0.63 | 2.69E-02 |  | ischemic brain damage | |
| P25786 | Proteasome subunit alpha type-1 | ↓ | 0.37 | 3.01E-02 |  | Bipolar Disorder | |
| O75131 | Copine-3 | ↑ | 1.84 | 3.18E-02 |  | Creutzfeldt-Jakob disease | |
| Q24JP5 | Transmembrane protein 132A | ↓ | 0.45 | 3.32E-02 |  | Synaptic Reorganization | |
| P59998 | Actin-related protein 2/3 complex subunit 4 | ↓ | 0.55 | 3.32E-02 |  | Brain-derived neurotrophic factor | |
| O95497 | Pantetheinase | ↓ | 0.58 | 3.44E-02 | increased in MDD | |  |
| P03950 | Angiogenin | ↑ | 3.17 | 3.90E-02 |  | Brain-derived neurotrophic factor | |
| P49418 | Amphiphysin | ↑ | 11.00 | 4.18E-02 |  | Schizophrenia | |
| Q695T7 | Sodium-dependent neutral amino acid transporter B(0)AT1 | ↑ | 2.00 | 4.18E-02 |  |  | |
| Q06828 | Fibromodulin | ↓ | 0.17 | 4.18E-02 |  |  | |
| P12318 | Low affinity immunoglobulin gamma Fc region receptor II-a | ↓ | 0.37 | 4.38E-02 |  |  | |
| Q10588 | ADP-ribosyl cyclase/cyclic ADP-ribose hydrolase 2 | ↓ | 0.55 | 4.97E-02 |  | synaptic depression in hippocampus | |

**Supplemental material 6 Differential proteins identified on week 2 in nonrespondent group in the second trial**

| Accession | Protein name | Trend | FC | P | related to major depressive disorder | related to psychiatric disorders or processes | |
| --- | --- | --- | --- | --- | --- | --- | --- |
| Q5JZY3 | Ephrin type-A receptor 10 | ↓ | 0.46 | 1.05E-03 |  | synaptic transmission | |
| Q9UBG0 | C-type mannose receptor 2 | ↓ | 0.61 | 1.60E-03 |  |  | |
| Q9NPY3 | Complement component C1q receptor | ↓ | 0.41 | 1.64E-03 |  | Alzheimer’s Disease | |
| Q6FHJ7 | Secreted frizzled-related protein 4 | ↓ | 0.51 | 2.45E-03 |  | Brain-derived neurotrophic factor | |
| P22748 | Carbonic anhydrase 4 | ↓ | 0.50 | 2.56E-03 |  |  | |
| P38571 | Lysosomal acid lipase/cholesteryl ester hydrolase | ↓ | 0.35 | 3.00E-03 |  |  | |
| P61457 | Pterin-4-alpha-carbinolamine dehydratase | ↓ | 0.65 | 3.24E-03 |  |  | |
| P00533 | Epidermal growth factor receptor | ↑ | 1.75 | 3.86E-03 |  | EGFR, ERBB signaling | |
| O75487 | Glypican-4 | ↓ | 0.53 | 4.64E-03 |  | Synaptic organization | |
| Q9NY97 | N-acetyllactosaminide beta-1,3-N-acetylglucosaminyltransferase 2 | ↓ | 0.53 | 4.76E-03 |  |  | |
| Q9H1U4 | Multiple epidermal growth factor-like domains protein 9 | ↓ | 0.32 | 6.10E-03 |  | Nervous system development | |
| P35270 | Sepiapterin reductase | ↓ | 0.59 | 6.26E-03 | increased in MDD drug treatment | |  |
| Q9BXP8 | Pappalysin-2 | ↓ | 0.63 | 8.41E-03 |  |  | |
| P15848 | Arylsulfatase B | ↓ | 0.54 | 8.47E-03 |  | Nervous system development | |
| P12277 | Creatine kinase B-type | ↑ | 1.71 | 8.65E-03 |  | Brain damage | |
| Q06481 | Amyloid-like protein 2 | ↓ | 0.58 | 8.66E-03 |  | Synaptic inhibition | |
| P24593 | Insulin-like growth factor-binding protein 5 | ↑ | 3.25 | 9.99E-03 |  | growth and differentiation of neuroblastoma cells | |
| P01225 | Follitropin subunit beta | ↓ | 0.43 | 1.07E-02 |  |  | |
| Q13938 | Calcyphosin | ↑ | 2.33 | 1.07E-02 |  |  | |
| Q5VW32 | BRO1 domain-containing protein BROX | ↓ | 0.52 | 1.15E-02 |  |  | |
| Q9Y6X5 | Bis(5'-adenosyl)-triphosphatase ENPP4 | ↓ | 0.12 | 1.19E-02 |  |  | |
| P29992 | Guanine nucleotide-binding protein subunit alpha-11 | ↓ | 0.55 | 1.19E-02 |  | The hypothalamic–pituitary–adrenal axis | |
| Q9HCM3 | UPF0606 protein KIAA1549 | ↓ | 0.42 | 1.19E-02 |  |  | |
| O75074 | Low-density lipoprotein receptor-related protein 3 | ↓ | 0.36 | 1.28E-02 |  | Synaptic plasticity | |
| Q9UBI6 | Guanine nucleotide-binding protein G(I)/G(S)/G(O) subunit gamma-12 | ↓ | 0.52 | 1.33E-02 |  |  | |
| P21333 | Filamin-A | ↑ | 1.58 | 1.62E-02 |  | Periventricular Nodular Heterotopia and Epilepsy | |
| P04275 | von Willebrand factor | ↓ | 0.57 | 1.65E-02 |  |  | |
| P78417 | Glutathione S-transferase omega-1 | ↓ | 0.59 | 1.93E-02 |  | BDNF | |
| Q16832 | Discoidin domain-containing receptor 2 | ↓ | 0.64 | 1.99E-02 |  |  | |
| Q8IX04 | Ubiquitin-conjugating enzyme E2 variant 3 | ↓ | 0.56 | 2.07E-02 |  | Synapse formation | |
| Q15746 | Myosin light chain kinase, smooth muscle | ↑ | 3.13 | 2.20E-02 |  | Notch Signaling | |
| P06310 | Immunoglobulin kappa variable 2-30 | ↑ | 2.00 | 2.21E-02 |  |  | |
| Q9NZU0 | Leucine-rich repeat transmembrane protein FLRT3 | ↓ | 0.51 | 2.29E-02 |  | Neurite outgrowth | |
| P58546 | Myotrophin | ↑ | 4.33 | 2.29E-02 |  |  | |
| Q16610 | Extracellular matrix protein 1 | ↑ | 2.56 | 2.32E-02 |  |  | |
| Q9BQT9 | Calsyntenin-3 | ↓ | 0.45 | 2.39E-02 |  | Alzheimer’s Disease | |
| Q9Y490 | Talin-1 | ↑ | 2.78 | 2.40E-02 |  |  | |
| P27824 | Calnexin | ↑ | 2.05 | 2.51E-02 |  | Neuroinflammation and myelin destruction | |
| Q9HC84 | Mucin-5B | ↑ | 4.93 | 2.53E-02 |  |  | |
| P05026 | Sodium/potassium-transporting ATPase subunit beta-1 | ↓ | 0.55 | 2.57E-02 |  |  | |
| P51148 | Ras-related protein Rab-5C | ↓ | 0.64 | 2.57E-02 |  |  | |
| Q9H6B4 | CXADR-like membrane protein | ↑ | 1.51 | 2.66E-02 |  |  | |
| Q13621 | Solute carrier family 12 member 1 | ↓ | 0.48 | 2.79E-02 |  |  | |
| P35754 | Glutaredoxin-1 | ↑ | 1.54 | 2.95E-02 |  | Parkinson's Disease | |
| Q96KN2 | Beta-Ala-His dipeptidase | ↓ | 0.33 | 3.01E-02 |  | Anderson-Fabry disease | |
| P32320 | Cytidine deaminase | ↑ | 16.50 | 3.02E-02 |  |  | |
| O15031 | Plexin-B2 | ↓ | 0.58 | 3.10E-02 |  | Synaptic Reorganization | |
| P06731 | Carcinoembryonic antigen-related cell adhesion molecule 5 | ↑ | 1.74 | 3.43E-02 |  |  | |
| P09543 | 2',3'-cyclic-nucleotide 3'-phosphodiesterase | ↓ | 0.40 | 3.73E-02 |  | Schizophrenia | |
| Q7Z5N4 | Protein sidekick-1 | ↓ | 0.67 | 3.80E-02 |  | Synaptic Reorganization | |
| P08183 | ATP-dependent translocase ABCB1 | ↓ | 0.47 | 3.90E-02 |  |  | |
| Q9Y274 | Type 2 lactosamine alpha-2,3-sialyltransferase | ↓ | 0.56 | 3.93E-02 |  | Inflammatory cytokines | |
| Q8N4F0 | BPI fold-containing family B member 2 | ↑ | 12.60 | 4.05E-02 |  |  | |
| O60218 | Aldo-keto reductase family 1 member B10 | ↑ | ∞ | 4.14E-02 |  |  | |
| Q02083 | N-acylethanolamine-hydrolyzing acid amidase | ↓ | 0.17 | 4.18E-02 |  | Anxiety | |
| P16562 | Cysteine-rich secretory protein 2 | ↓ | 0.33 | 4.45E-02 |  |  | |
| Q9Y5Y6 | Suppressor of tumorigenicity 14 protein | ↓ | 0.39 | 4.45E-02 |  |  | |
| O00401 | Neural Wiskott-Aldrich syndrome protein | ↓ | 0.48 | 4.75E-02 |  | Alzheimer’s Disease | |
| Q9NZV1 | Cysteine-rich motor neuron 1 protein | ↓ | 0.57 | 4.77E-02 |  | Neurogenesis | |
| Q6UWI4 | Protein shisa-2 homolog | ↑ | 1.61 | 4.84E-02 |  |  | |

**Supplemental material 7 Differential proteins identified at week 12 in the respondent group in the second trial**

| Accession | Protein name | Trend | FC | P | related to major depressive disorder | related to psychiatric disorders or processes |
| --- | --- | --- | --- | --- | --- | --- |
| Q92896 | Golgi apparatus protein 1 | ↓ | 0.66 | 7.69E-03 |  | Amyotrophic lateralizing sclerosis |
| Q96MU8 | Kremen protein 1 | ↓ | 0.43 | 1.06E-02 |  | Central nervous system functioning |
| P59998 | Actin-related protein 2/3 complex subunit 4 | ↓ | 0.48 | 1.50E-02 | Differentially expressed in hippocampus of MDD mouse |  |
| P47756 | F-actin-capping protein subunit beta | ↓ | 0.54 | 1.81E-02 | Differentially expressed in platelet of MDD subjects |  |
| P84077 | ADP-ribosylation factor 1 | ↓ | 0.60 | 1.84E-02 |  | Synaptic plasticity |
| P29966 | Myristoylated alanine-rich C-kinase substrate | ↓ | 0.60 | 2.26E-02 | Differentially expressed in brain of MDD subjects |  |
| Q96PC5 | Melanoma inhibitory activity protein 2 | ↑ | 3.00 | 2.29E-02 |  |  |
| Q14165 | Malectin | ↑ | 4.50 | 2.48E-02 |  | Cerebral palsy |
| Q6P9A2 | Polypeptide N-acetylgalactosaminyltransferase 18 | ↓ | 0.48 | 2.58E-02 |  |  |
| O60547 | GDP-mannose 4,6 dehydratase | ↓ | 0.59 | 2.94E-02 |  |  |
| Q99536 | Synaptic vesicle membrane protein VAT-1 homolog | ↓ | 0.45 | 3.08E-02 |  |  |
| Q86TH1 | ADAMTS-like protein 2 | ↓ | 0.59 | 3.19E-02 |  |  |
| Q09328 | Alpha-1,6-mannosylglycoprotein 6-beta-N-acetylglucosaminyltransferase A | ↓ | 0.46 | 3.44E-02 |  | Susceptibility gene of schizophrenia |
| P49788 | Retinoic acid receptor responder protein 1 | ↓ | 0.65 | 3.47E-02 |  |  |
| Q8NI99 | Angiopoietin-related protein 6 | ↓ | 0.26 | 3.49E-02 |  |  |
| P21333 | Filamin-A | ↓ | 0.46 | 3.52E-02 |  | Axon regeneration |
| P13861 | cAMP-dependent protein kinase type II-alpha regulatory subunit | ↓ | 0.33 | 3.68E-02 |  |  |
| Q9BQT9 | Calsyntenin-3 | ↓ | 0.33 | 3.68E-02 |  | Alzheimer's disease |
| P16284 | Platelet endothelial cell adhesion molecule | ↑ | 1.57 | 3.68E-02 | SSRI therpy of MDD subjects |  |
| P19801 | Amiloride-sensitive amine oxidase [copper-containing] | ↓ | 0.59 | 3.91E-02 |  |  |
| Q9NR34 | Mannosyl-oligosaccharide 1,2-alpha-mannosidase IC | ↓ | 0.50 | 0.04 |  |  |
| P52209 | 6-phosphogluconate dehydrogenase, decarboxylating | ↓ | 0.58 | 4.24E-02 |  |  |
| P62328 | Thymosin beta-4 | ↑ | 2.40 | 4.45E-02 | Recovery from depression |  |
| P20062 | Transcobalamin-2 | ↓ | 0.37 | 4.62E-02 |  |  |
| P17342 | Atrial natriuretic peptide receptor 3 | ↓ | 0.64 | 4.87E-02 | Neuroprotection |  |

**Supplemental material 8 Differential proteins identified at week 12 in the nonrespondent group in the second trial**

| Accession | Protein name | Trend | FC | P | Related to major depressive disorder | Related to psychiatric disorders or processes |
| --- | --- | --- | --- | --- | --- | --- |
| Q6ISS4 | Leukocyte-associated immunoglobulin-like receptor 2 | ↓ | 0.61 | 5.77E-03 |  |  |
| P10321 | HLA class I histocompatibility antigen, C alpha chain | ↑ | 1.50 | 5.77E-03 | Interferon pathway |  |
| P62714 | Serine/threonine-protein phosphatase 2A catalytic subunit beta isoform | ↑ | 3.75 | 6.74E-03 | Improve the depression-like phenotype in mice |  |
| Q9UJJ9 | N-acetylglucosamine-1-phosphotransferase subunit gamma | ↑ | 4.67 | 6.74E-03 |  |  |
| O43567 | E3 ubiquitin-protein ligase RNF13 | ↓ | 0.62 | 7.78E-03 |  |  |
| Q5JZY3 | Ephrin type-A receptor 10 | ↓ | 0.48 | 9.11E-03 |  | Ephrin signaling |
| Q9H0R4 | Haloacid dehalogenase-like hydrolase domain-containing protein 2 | ↑ | 2.00 | 9.53E-03 | Neuroprotection |  |
| Q9Y490 | Talin-1 | ↑ | 1.94 | 1.16E-02 |  |  |
| Q8N257 | Histone H2B type 3-B | ↑ | 3.15 | 1.25E-02 |  |  |
| O60911 | Cathepsin L2 | ↑ | 2.33 | 1.30E-02 |  |  |
| O60704 | Protein-tyrosine sulfotransferase 2 | ↓ | 0.37 | 1.30E-02 |  |  |
| P55786 | Puromycin-sensitive aminopeptidase | ↑ | 6.00 | 1.46E-02 |  | Neuroprotection in Alzheimer's disease |
| Q9UGM3 | Deleted in malignant brain tumors 1 protein | ↑ | 3.50 | 1.50E-02 |  | Neuroglioma |
| P36543 | V-type proton ATPase subunit E 1 | ↑ | 1.95 | 1.58E-02 |  | Alzheimer's disease |
| P25787 | Proteasome subunit alpha type-2 | ↑ | 2.14 | 1.61E-02 |  | Neuroglioma |
| Q6FHJ7 | Secreted frizzled-related protein 4 | ↓ | 0.55 | 1.74E-02 |  | PI3K / AKT signaling |
| P14618 | Pyruvate kinase PKM | ↑ | 1.89 | 1.81E-02 |  | Inhibition of medulloblastoma |
| Q9UGN4 | CMRF35-like molecule 8 | ↓ | 0.65 | 1.86E-02 |  |  |
| P42702 | Leukemia inhibitory factor receptor | ↓ | 0.62 | 2.20E-02 | Neuroprotection |  |
| O95841 | Angiopoietin-related protein 1 | ↑ | 5.00 | 2.24E-02 |  | Integrin binding |
| O60701 | UDP-glucose 6-dehydrogenase | ↑ | 2.83 | 0.02 |  | Epileptic encephalopathy |
| P31948 | Stress-induced-phosphoprotein 1 | ↑ | 5.50 | 2.39E-02 |  | Neuro-Behçet's disease (NBD) |
| P28676 | Grancalcin | ↑ | 2.57 | 2.43E-02 |  |  |
| Q96RM1 | Small proline-rich protein 2F | ↑ | 1.83 | 2.58E-02 |  |  |
| P28070 | Proteasome subunit beta type-4 | ↑ | 1.73 | 2.93E-02 |  | Nerve inflammation |
| Q13765 | Nascent polypeptide-associated complex subunit alpha | ↑ | 3.25 | 2.94E-02 |  | Traumatic brain injury |
| P04439 | HLA class I histocompatibility antigen, A alpha chain | ↑ | 1.78 | 2.94E-02 | Depression susceptibility gene |  |
| O95171 | Sciellin | ↑ | 1.83 | 2.97E-02 |  |  |
| Q14703 | Membrane-bound transcription factor site-1 protease | ↑ | 2.00 | 3.01E-02 |  |  |
| Q9Y4D7 | Plexin-D1 | ↓ | 0.64 | 3.01E-02 |  | Synapse formation |
| Q14314 | Fibroleukin | ↑ | 2.23 | 3.17E-02 |  | Neuroprotection |
| P52907 | F-actin-capping protein subunit alpha-1 | ↑ | 2.43 | 3.19E-02 |  | Neuroblastoma |
| P20810 | Calpastatin | ↑ | 1.57 | 3.27E-02 |  |  |
| P25786 | Proteasome subunit alpha type-1 | ↑ | 3.17 | 3.32E-02 |  | Parkinson's disease |
| P17655 | Calpain-2 catalytic subunit | ↑ | #DIV/0! | 3.32E-02 | Nerve growth related genes |  |
| P30740 | Leukocyte elastase inhibitor | ↑ | 2.34 | 3.33E-02 | Inflammatory and autoimmune markers |  |
| Q8IX04 | Ubiquitin-conjugating enzyme E2 variant 3 | ↓ | 0.60 | 3.54E-02 |  | Axon termination and synapse formation |
| Q96A08 | Histone H2B type 1-A | ↑ | 1.94 | 3.62E-02 |  |  |
| P09972 | Fructose-bisphosphate aldolase C | ↑ | 1.92 | 3.64E-02 |  |  |
| P35221 | Catenin alpha-1 | ↑ | #DIV/0! | 3.68E-02 | Improvement of depressive behaviors |  |
| Q5IJ48 | Protein crumbs homolog 2 | ↓ | 0.66 | 3.71E-02 |  |  |
| P01040 | Cystatin-A | ↑ | 1.53 | 3.72E-02 |  |  |
| P24593 | Insulin-like growth factor-binding protein 5 | ↑ | 4.25 | 3.90E-02 |  | Neuroblastoma |
| Q9UBG3 | Cornulin | ↑ | 1.56 | 3.94E-02 |  |  |
| P04632 | Calpain small subunit 1 | ↑ | 2.32 | 3.96E-02 |  | Axonal injury |
| P15104 | Glutamine synthetase | ↑ | 3.19 | 0.04 | Neuroprotection |  |
| P29692 | Elongation factor 1-delta | ↑ | #DIV/0! | 4.14E-02 |  |  |
| Q08629 | Testican-1 | ↑ | 1.89 | 4.15E-02 |  | Alzheimer's disease |
| P27482 | Calmodulin-like protein 3 | ↑ | 1.61 | 4.24E-02 |  |  |
| P02545 | Prelamin-A/C | ↑ | 5.29 | 4.26E-02 |  | Charcot-Marie-Tooth disease |
| P32926 | Desmoglein-3 | ↑ | 2.05 | 4.41E-02 |  |  |
| P34932 | Heat shock 70 kDa protein 4 | ↑ | 3.00 | 4.43E-02 |  |  |
| Q9H1U4 | Multiple epidermal growth factor-like domains protein 9 | ↓ | 0.53 | 4.43E-02 |  |  |
| P08311 | Cathepsin G | ↑ | 5.11 | 4.44E-02 |  |  |
| P36952 | Serpin B5 | ↑ | 17.33 | 4.58E-02 |  |  |
| O43488 | Aflatoxin B1 aldehyde reductase member 2 | ↑ | 1.69 | 4.69E-02 |  | Neurodegenerative diseases |
| Q96HE7 | ERO1-like protein alpha | ↑ | 2.30 | 4.78E-02 |  |  |
| Q9HC84 | Mucin-5B | ↑ | 3.48 | 4.93E-02 |  |  |
| P49720 | Proteasome subunit beta type-3 | ↑ | 3.80 | 4.98E-02 |  |  |


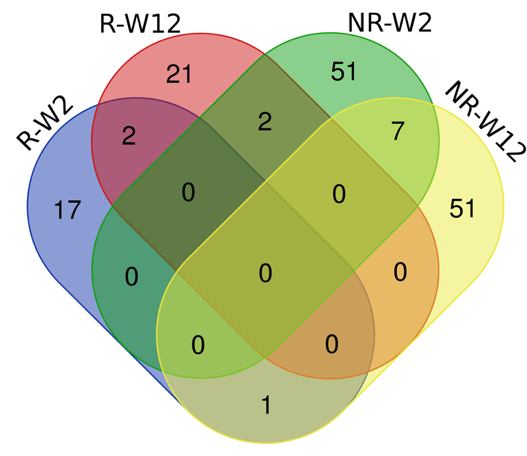


**Supplemental material 9 Overlap evaluation of the differential proteins identified at different time points and groups in the second trial.** (*P* < 0.05 and fold change ≥ 1.5 or ≤ 0.67).


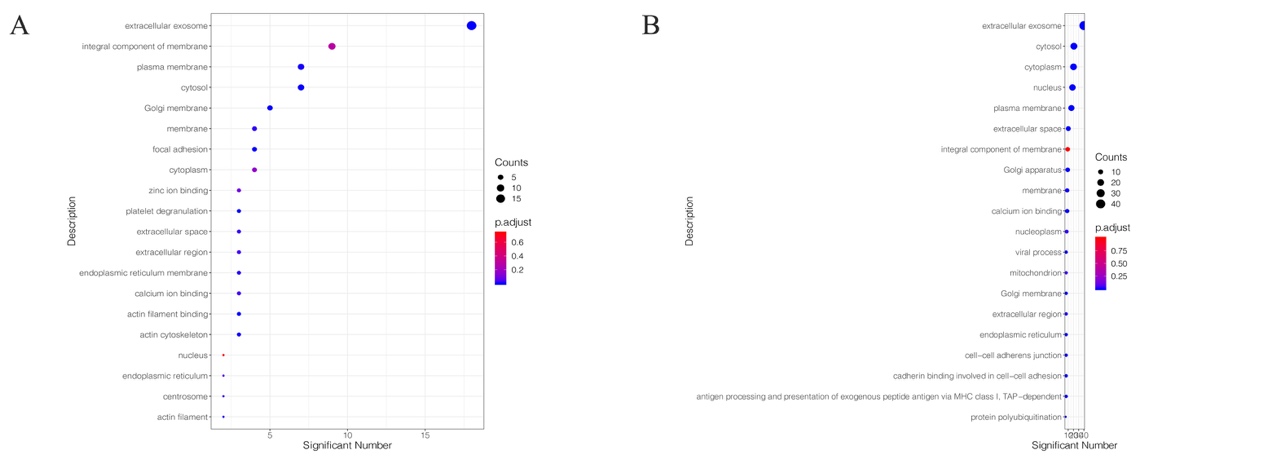


**Supplemental material 10 Gene ontology (GO) analysis of urinary differential proteins at week 12 in the second trial.** GO analysis of the differential proteins was performed to study the specific molecular functions (MF), biological processes (BP), and cellular components (CC) (the top 20 counts are shown, adjusted p < 0.05 with BH correction). The criteria of *P* < 0.05 and a fold change ≥ 1.5 or ≤ 0.67 were applied for screening differential proteins. (A) GO analysis on week 12 in respondent group. (B) GO analysis on week 12 in nonrespondent group.


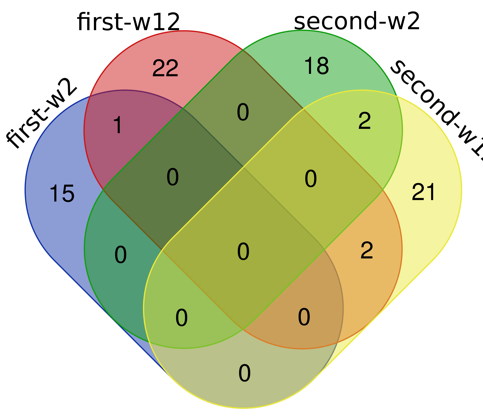


**Supplemental material 11 Venn diagram of differential proteins at different time points in escitalopram respondent groups between two trials.** (*P* < 0.05 and fold change ≥ 1.5 or ≤ 0.67).
